# Geochemical and Visual Indicators of Hydrothermal Fluid Flow through a Sediment-Hosted Volcanic Ridge in the Central Bransfield Basin (Antarctica)

**DOI:** 10.1371/journal.pone.0054686

**Published:** 2013-01-24

**Authors:** Alfred Aquilina, Douglas P. Connelly, Jon T. Copley, Darryl R. H. Green, Jeffrey A. Hawkes, Laura E. Hepburn, Veerle A. I. Huvenne, Leigh Marsh, Rachel A. Mills, Paul A. Tyler

**Affiliations:** 1 Ocean and Earth Sciences, University of Southampton Waterfront Campus, European Way, Southampton, United Kingdom; 2 Marine Geoscience, National Oceanography Centre, European Way, Southampton, United Kingdom; Utrecht University, The Netherlands

## Abstract

In the austral summer of 2011 we undertook an investigation of three volcanic highs in the Central Bransfield Basin, Antarctica, in search of hydrothermal activity and associated fauna to assess changes since previous surveys and to evaluate the extent of hydrothermalism in this basin. At Hook Ridge, a submarine volcanic edifice at the eastern end of the basin, anomalies in water column redox potential (E_h_) were detected close to the seafloor, unaccompanied by temperature or turbidity anomalies, indicating low-temperature hydrothermal discharge. Seepage was manifested as shimmering water emanating from the sediment and from mineralised structures on the seafloor; recognisable vent endemic fauna were not observed. Pore fluids extracted from Hook Ridge sediment were depleted in chloride, sulfate and magnesium by up to 8% relative to seawater, enriched in lithium, boron and calcium, and had a distinct strontium isotope composition (^87^Sr/^86^Sr  = 0.708776 at core base) compared with modern seawater (^87^Sr/^86^Sr ≈0.70918), indicating advection of hydrothermal fluid through sediment at this site. Biogeochemical zonation of redox active species implies significant moderation of the hydrothermal fluid with in situ diagenetic processes. At Middle Sister, the central ridge of the Three Sisters complex located about 100 km southwest of Hook Ridge, small water column E_h_ anomalies were detected but visual observations of the seafloor and pore fluid profiles provided no evidence of active hydrothermal circulation. At The Axe, located about 50 km southwest of Three Sisters, no water column anomalies in E_h_, temperature or turbidity were detected. These observations demonstrate that the temperature anomalies observed in previous surveys are episodic features, and suggest that hydrothermal circulation in the Bransfield Strait is ephemeral in nature and therefore may not support vent biota.

## Introduction

Hydrothermal circulation through young ocean crust is a ubiquitous phenomenon leading to significant metal enrichment at the seafloor [1] and supporting diverse and unique fauna [2]. However, the Antarctic region has not been widely explored for hydrothermal activity, mainly because of its remoteness and the hostile environment associated with high latitudes. Nevertheless, evidence for hydrothermal activity in the Southern Ocean has been established for more than a decade, primarily based on observations of chemical anomalies in the water column [3], [4], [5]. Because of the relative isolation of Antarctic hydrothermal fields from other hydrothermal systems and the mid-ocean ridge system, their occurrence has important implications for the distribution and evolution of vent-associated fauna. Indeed, this is supported by the recent discovery of unique chemosynthetic communities associated with hydrothermal vents on the East Scotia Ridge in the Southern Ocean [6].

**Figure 1:**
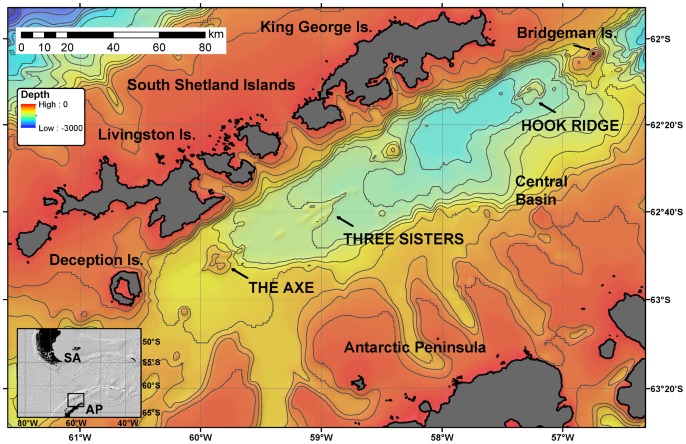
Regional map of the Bransfield Strait. Location map of the Bransfield Strait in the Southern Ocean with bathymetry of the central basin, showing the location of the submarine volcanic edifices Hook Ridge, Three Sisters and The Axe, and the subaerial volcanoes Deception and Bridgeman Islands. Map constructed using GEBCO bathymetry.

The Bransfield Strait is a marginal basin located between the Northern Antarctic Peninsula and the South Shetland Islands (Figure 1), formed by rifting of continental crust [7] at spreading rates of 2.5–7.5 mm yr^−1^ [8]. It has a maximum width of 80 km and a length of more than 400 km between Small Island in the southwest and Clarence Island in the northeast. The basin is divided into the western, central and eastern sub-basins by the sub-aerial volcanoes Deception and Bridgeman Islands, located 200 km apart along the central rifting axis (Figure 1). The central basin is characterised by high sedimentation rates of up to1.8 mm yr^−1^ [9], [10] and a sediment cover thickness of up to 3.3 km [11]. The bathymetry of the central basin is relatively uniform, with the exception of a number of ridges on the seafloor [12]. On the central rift axis, several volcanic edifices rise above the seafloor, including Hook Ridge (summit at 1050 m water depth), the Three Sisters complex (1310 m water depth) and The Axe (also known as Edifice A; 1025 m water depth) (Figure 1).

The first reported indications of hydrothermal activity in the Bransfield Strait derived from measurements of water column anomalies, namely excess ^3^He [13], [14] and elevated concentrations of CH_4_ [15] and Mn [4], [14]. These were supported by reports of thermally-altered sediments [14], [16], and the presence of silicified patches and hydrothermal precipitates at Hook Ridge and Three Sisters [15], [17]. Additionally, a siboglinid polychaete (*Sclerolinum* sp.) was observed in hydrothermally-influenced sediment cores characterised by hydrogen sulfide fluxes of 0.03 and 0.05 mol m^−2^ yr^−1^ [18]. *Sclerolinum* species, however, may be considered organic-enrichment opportunists, as they are not exclusive to hydrothermal vent environments [19], [20], and vent-typical organisms have not been found in the region, arguably because of low sulfide availability [18].

In the austral summer of 2011, on board the RRS *James Cook*, we carried out a survey of the Central Bransfield Basin in an attempt to locate and sample sites of hydrothermal venting and associated fauna. We focused our investigation on Hook Ridge and Middle Sister, the central ridge of the Three Sisters complex, where hydrothermal activity had been previously documented [21]. In addition, we aimed to investigate the water column in the region of The Axe, a submarine volcanic edifice which had not been previously studied for hydrothermal activity. The motivation for our exploration was to (i) identify the current location of hydrothermal discharge, (ii) sample sediments from hydrothermally-influenced areas to assess the associated chemical fluxes and (iii) examine fauna associated with potential hydrothermal activity in this isolated biogeographic province. Our extensive water column surveys in the central basin were followed by visual observations of hydrothermal fluid discharge at the seafloor on Hook Ridge. These observations were corroborated by anomalies in the geochemical composition of pore fluids. In contrast, there was no evidence of hydrothermal activity at Middle Sister and The Axe. A recognisable vent-endemic fauna, or an increase in abundance of fauna, was not observed at any of the surveyed areas.

## Materials and Methods

### Ethics Statement

All necessary permits (numbers S5-4/2010) were obtained for the described field study from the South Georgia and South Sandwich Islands Government, in accordance with the Antarctic Act 1994 and the Antarctic Regulations 1995.

### Bathymetric and Water Column Surveys

**Figure 2:**
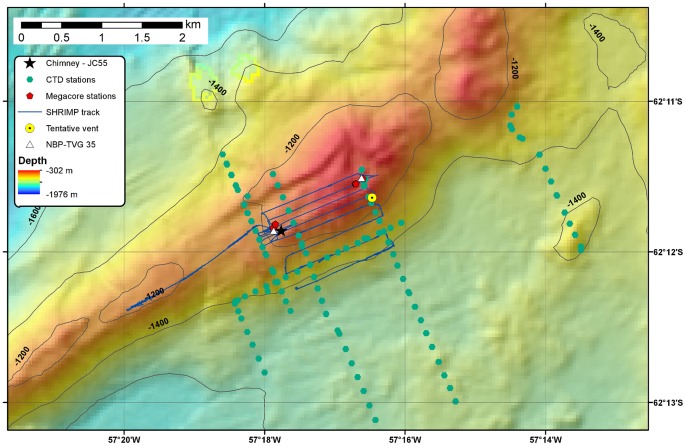
Detailed bathymetric map of Hook Ridge. Map showing JC55 SHRIMP and CTD tracks and coring locations. Vent sites inferred from previous studies (denoted by yellow circle and white triangle) are included for comparison.

**Figure 3:**
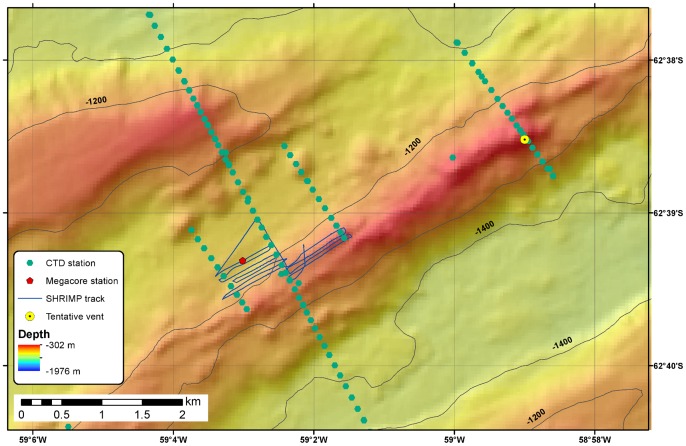
Detailed bathymetry of the Middle Sister ridge. Map showing JC55 survey tracks and coring stations at Middle Sister. Previously-inferred vent location is shown for comparison (yellow circle).

**Figure 4:**
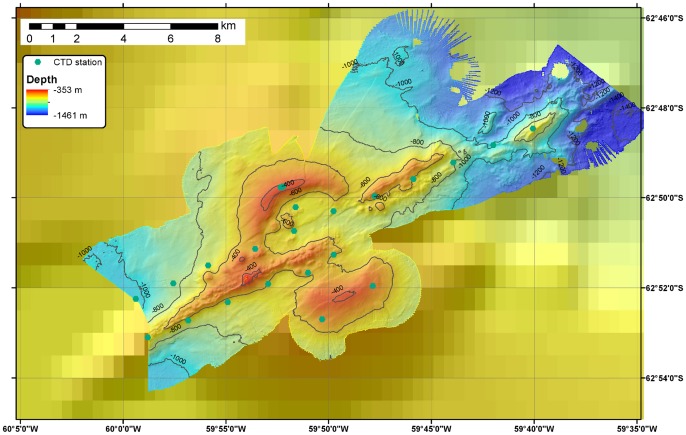
Detailed bathymetry of The Axe. Map showing the JC55 CTD survey track in the water column above The Axe. In spite of extensive surveys, no anomalies in temperature, E_h_ or LSS were detected.

On the RRS *James Cook* 055, seafloor mapping used a hull-mounted Kongsberg-Simrad EM120 multibeam echosounder. Data processing was carried out with the Caraibes software (IFREMER). Water column surveys were conducted with a SeaBird 911 conductivity, temperature, depth (CTD) system equipped with a bespoke oxidation-reduction potential (E_h_) sensor and a light scattering sensor (LSS). The E_h_ sensor, developed by Ko-ichi Nakamura (National Institute of Advanced Industrial Science and Technology, Tsukuba, Japan), has previously been used to detect reducing species in hydrothermal plumes in areas including the East Scotia Ridge [6], the Cayman Trough [22] and the Loihi seamount [23]. The CTD instrument package was deployed in single vertical casts and in tow-yo mode, where the equipment is lowered through a portion of the water column with the ship stationary and subsequently raised while the ship moves slowly along a survey line. At Hook Ridge, a total of five tow-yo transects were carried out, with additional individual vertical profiles taken in the region of negative E_h_ anomalies south of the ridge. At Three Sisters four tow-yo transects and several vertical profiles were undertaken, and at The Axe twenty-two full-depth CTD profiles were measured. CTD stations, defined as inflections in the case of tow-yo profiles, are shown on the bathymetric maps in Figures 2–4.

### Seafloor Observations

A lowered camera system, Seabed High Resolution Imaging Platform (SHRIMP), with down-looking and oblique video cameras was used to survey the seafloor. Two parallel lasers, mounted 0.1 m apart, provided a scale in camera images for assessments of faunal abundance. Based on information collected during CTD surveys, SHRIMP imaged the seafloor at the centre and on the southern slope of Hook Ridge. A series of contour-parallel survey lines were followed, stepping up-slope on the southern face of the ridge, continuing this pattern across the ridge crest (Figure 2). At Three Sisters, SHRIMP surveyed the northern slope of the central ridge, undertaking a series of contour-parallel lines moving down-slope and subsequently moving south to the crest of the ridge to undertake a further series of lines southeast of the original survey grid (Figure 3). Because of the absence of water column signals and due to time constraints, SHRIMP was not deployed at The Axe.

### Sample Collection and Handling

Sediment cores were retrieved using a Bowers & Connelly megacorer equipped with 10 cm diameter polycarbonate tubes; cores were obtained from Hook Ridge (stations MC7 and MC16) and Middle Sister (station MC24) (Figures 2 and 3), and from reference stations situated at similar water depth on the Antarctic Peninsula shelf, about 20 km south of Hook Ridge (MC2 and MC3). Geographical co-ordinates and water depths of coring stations are listed in Table 1. Megacores were not retrieved from The Axe because of shallow sediment cover.

**Table 1:** Geographical co-ordinates and water depth at the JC55 coring stations.

Station – Core ID	Latitude (°S)	Longitude (°W)	Water depth (m)
Reference – MC02	62.3842	57.2444	1150
Reference – MC03	62.3842	57.2441	1148
Hook Ridge – MC07	62.1969	57.2975	1174
Hook Ridge – MC16	62.1924	57.2784	1040
Middle Sister – MC24	62.6552	59.0502	1311

Sediment temperature was measured by inserting a thermometer into the surface of the core soon after they arrived on deck [15], [21], [24]. Multicores were sampled for geochemistry at depth intervals of 1–2 cm in a constant temperature laboratory (∼4°C) in a glove bag under a nitrogen atmosphere on board the RRS *James Cook*. For headspace methane analysis, sediment sub-samples (∼3 ml) were taken using plastic syringes with their tip removed, transferred to a 20 ml glass vial and fixed with 5 ml of 1 M NaOH. The vials were sealed and shaken vigorously to release the dissolved gases into the headspace. Pore fluids were separated from the sediment matrix by centrifugation under a nitrogen atmosphere and subsequently filtered through a 0.2 µm membrane filter. Aliquots were used for onboard determination of dissolved chloride, sulfate, hydrogen sulfide and ammonium and total alkalinity. Additional aliquots were stored frozen (−20°C) for subsequent analysis of dissolved nitrate, and acidified to a pH <2 with HNO_3_ and stored chilled (∼4°C) for subsequent dissolved metal analysis. Residual sediments were freeze-dried on board and stored at room temperature for subsequent solid phase analysis at the shore-based laboratory.

### Analytical Procedures

Concentration of headspace methane was determined on-board by gas chromatography (Agilent 7890A) and converted to pore fluid concentrations by accounting for sediment porosity, which was calculated from the loss of water after drying the sediment at 60°C assuming densities of 2.6 and 1.025 g cm^−3^ for the sediment and pore fluid, respectively [18]. Hydrogen sulfide was fixed with zinc acetate and determined using standard photometric methods [25]. Ammonium concentrations were determined immediately after pore water extraction using standard photometric methods [26]. Concentrations of Cl^−^ and SO_4_^2−^ were determined by ion chromatography (Dionex ICS2500). The reproducibility of Cl^−^ and SO_4_^2−^ measurements, determined by replicate analyses of samples and IAPSO standard seawater (Ocean Scientific International Ltd, UK), is better than 2%. Total alkalinity was determined by titration against standardised 0.1 M HCl.

Nitrate concentrations were determined photometrically using a standard nutrient autoanalyser (QaAAtro, Seal Analytical). Calcium, lithium and boron concentrations were determined by inductively coupled plasma optical emission spectroscopy (ICP-OES; Perkin Elmer Optima 4300DV). Instrument precision, determined from five replicate measurements of the same sample, is better than 2% and measured concentrations of an artificial seawater standard (CRM-SW; High-Purity Standards) were within 1% of the recommended values.

The dissolved ^87^Sr/^86^Sr ratio in the deepest sample of core MC16 (22 cm depth) was determined using a TRITON *Plus* thermal ionization mass spectrometer (TIMS). Sub-samples (containing ∼ 1 µg of Sr) were evaporated to dryness, taken up in 3 M HNO_3_ and run through Sr-Spec columns. The purified Sr was then loaded onto out-gassed Ta filaments. Multiple analyses (x60) of NBS 987 standard yielded an average ^87^Sr/^86^Sr of 0.710246±0.000019 (2σ).

Bulk sediment samples were completely dissolved in acid mixtures using an established multi-step method [27]. The accuracy of the procedure was assessed by including certified reference materials (CRM) MAG-1 (US Geological Survey) and GSMS-2 [28]. Concentrations of metals including Mg and Ti were measured by ICP-OES (Perkin Elmer Optima 4300 DV) as 2000-fold dilutions in 0.6 M HCl. Calibration was done with a series of matrix-matched multi-element standards. Reported values were corrected for blank and instrument drift. Average measured values of replicate CRM digestions were within two standard deviations of the recommended value. The instrument precision, determined from five replicate measurements of each sample, is better than 2% for the elements presented here (i.e. Ti and Mg). The overall precision of the method, determined from replicate (x3) digestions of MAG-1 and GSMS-2, is better than 5%.

### Advection Rate Modelling

Fluid advection rates in Hook Ridge cores were estimated by fitting a one-dimensional transport-reaction model to the measured downcore [Cl^−^] profiles, assuming that molecular diffusion and advection are the only transport processes. Chloride was chosen because at low temperatures it behaves conservatively and, therefore, the reaction term can be omitted. Additionally, at Hook Ridge phase separation results in a hydrothermal end-member having low chlorinity [24]. If the uppermost [Cl^−^] is defined as C_o_ and the deepest [Cl^−^] is defined as C_base_, the solution to the advection-diffusion equation (C_z_) under steady-state conditions is [29]:
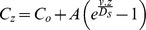


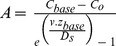
where ν is the advective pore fluid velocity, D_s_ is the whole sediment diffusion coefficient for Cl^−^ and *z* is depth below seafloor. The effective diffusion coefficient in seawater at 0°C (D_sw_) was determined from the diffusion coefficient at infinite dilution at 0°C (D° = 1.01×10^−5^ cm^2^ s^−1^; [30]) and adjusted for the difference in viscosity between seawater and an infinitely dilute solution (D_sw_ = 0.95×D°; [30]). Sediment tortuosity was estimated from the measured average sediment porosity (ø; 0.82 at MC7 and 0.79 at MC16) [31]:



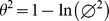
and this was used to correct D_sw_ at 0°C:



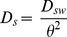



The calculated D_s_ at 0°C is 6.81×10^−6^ cm^2^ s^−1^ for MC7 and 6.49×10^−6^ cm^2^ s^−1^ for MC16. The best fit solution to the equation was obtained by minimizing the sum of the squares of the residual concentration difference between the observed and estimated [Cl^−^].

## Results

### Hydrographic and Water Column Characteristics

**Figure 5:**
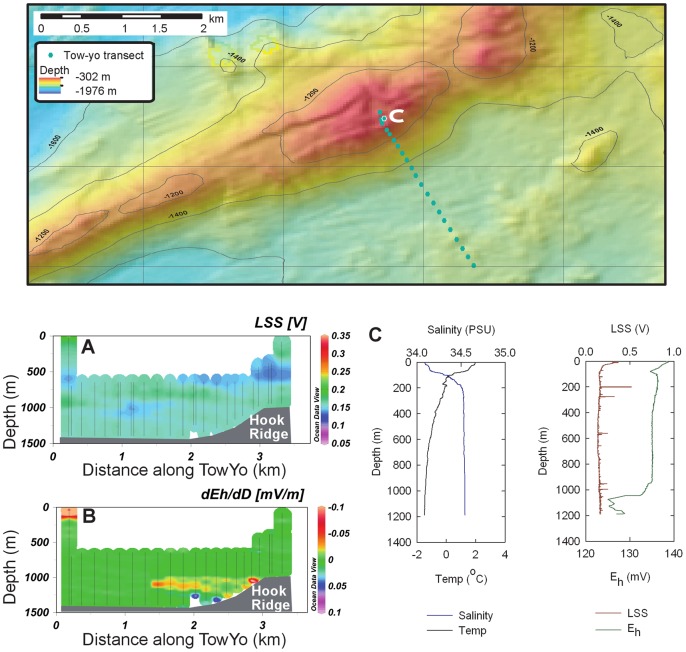
Characteristics of the water column near Hook Ridge. Contour plots of (A) LSS measurements and (B) E_h_ measurements measured along CTD tow-yo transect between 62°12.990′S, 57°15.282′W and 62°11.454′S, 57°16.614′W. (C) Depth profiles of salinity, temperature, LSS and Eh measured at 62°11.964′S 57°97.468′W. A near-bottom E_h_ anomaly of ∼ 10 mV was measured at ∼1100 m depth. The locations of the CTD stations are shown on the bathymetric map.

CTD surveys identified small negative anomalies in water column E_h_ at Hook Ridge close to the seafloor south of the main ridge, but there were no discernible changes in water column turbidity, temperature or salinity; this is in contrast to previous observations where temperature anomalies (of up to 0.3°C) and LSS anomalies were detected in the same region at Hook Ridge [21]. Contour plots of LSS and E_h_ sensor data are shown in Figures 5A and 5B. Because the E_h_ sensor is susceptible to instrument drift and strong hysteresis [32], [33], we use the depth derivative dE/dD to identify the location of E_h_ anomalies by rapidly changing E_h_ (Figure 5B). The sharpest E_h_ anomaly was measured in a CTD cast at 62°11.964′S 57°17.468′W at a depth of approximately 1100 m (the most reducing region identified in the tow-yo survey) and comprised a negative anomaly of ∼−10 mV (Figure 5C). This compares with an E_h_ anomaly of ∼−100 mV, measured using the same E_h_ sensor, in a high temperature hydrothermal buoyant plume in the Beebe Vent Field at the mid-Cayman Spreading Centre [22].

Water column CTD surveys in the areas of Middle Sister showed small negative E_h_ anomalies only over a small area of the ridge, while at The Axe no regions of anomalous E_h_, temperature or light scattering were identified.

### Seafloor Observations

**Figure 6:**
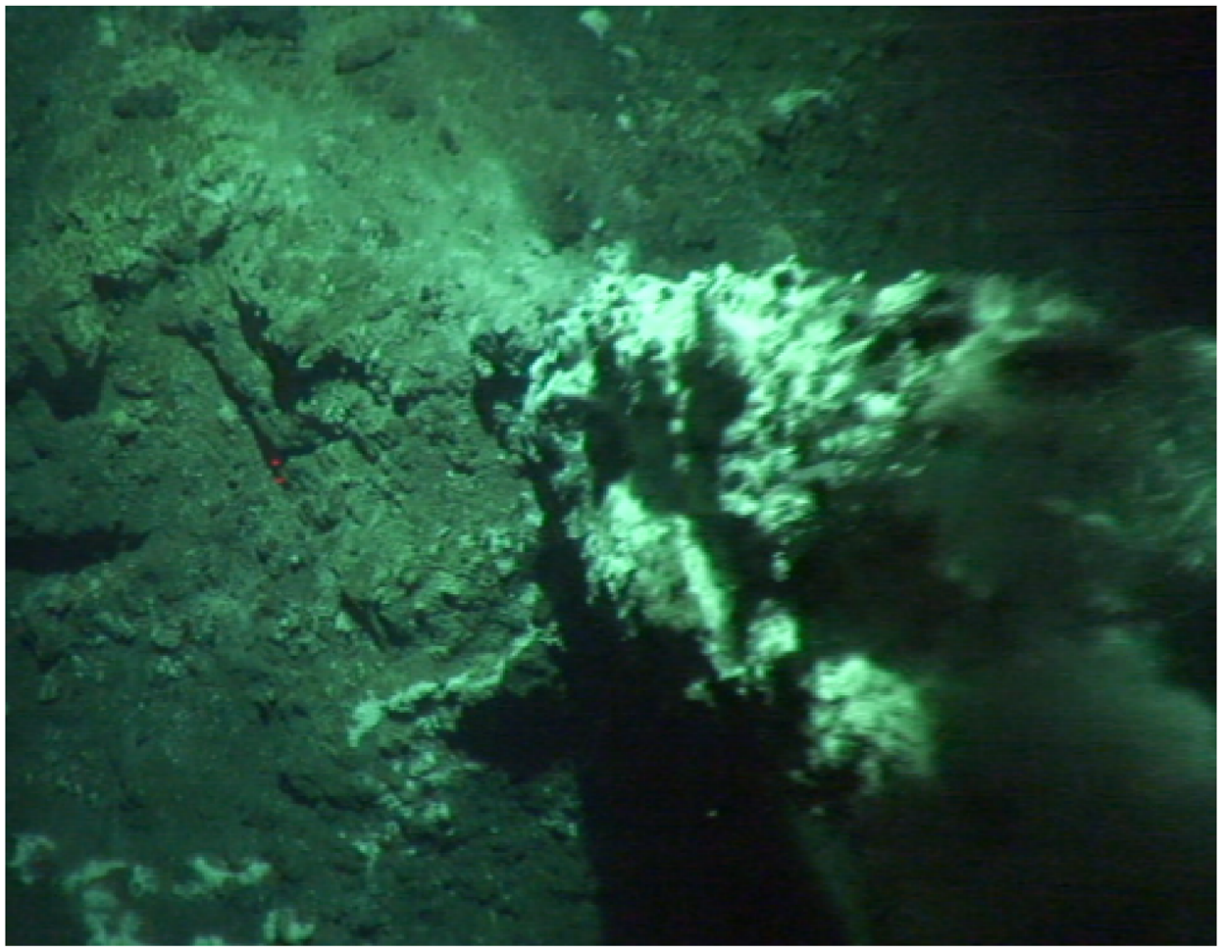
SHRIMP image of the seafloor at Hook Ridge. Image shows a ∼ 2 m-high chimney at a depth of ∼1200 m, location 62°11.858′S 57° 17.699′W, emanating hydrothermal fluid visible as shimmering water. The red laser dots visible in the image are 0.1 m apart.

The seafloor on the northern slope of Hook Ridge consisted of fine sediments with occasional rock outcrops. The crest of the ridge was marked by a graben-like depression with sheer walls of volcanic rock and hosted areas of rock outcrops, fine sediment and mineralised crust. Hydrothermal activity was observed around these sites in the form of shimmering water issuing from depressions in the seafloor and from small mineralised chimneys (Figure 6). White particles on the seafloor and shimmering water were also observed in the northeast corner of the crest survey area.

**Figure 7:**
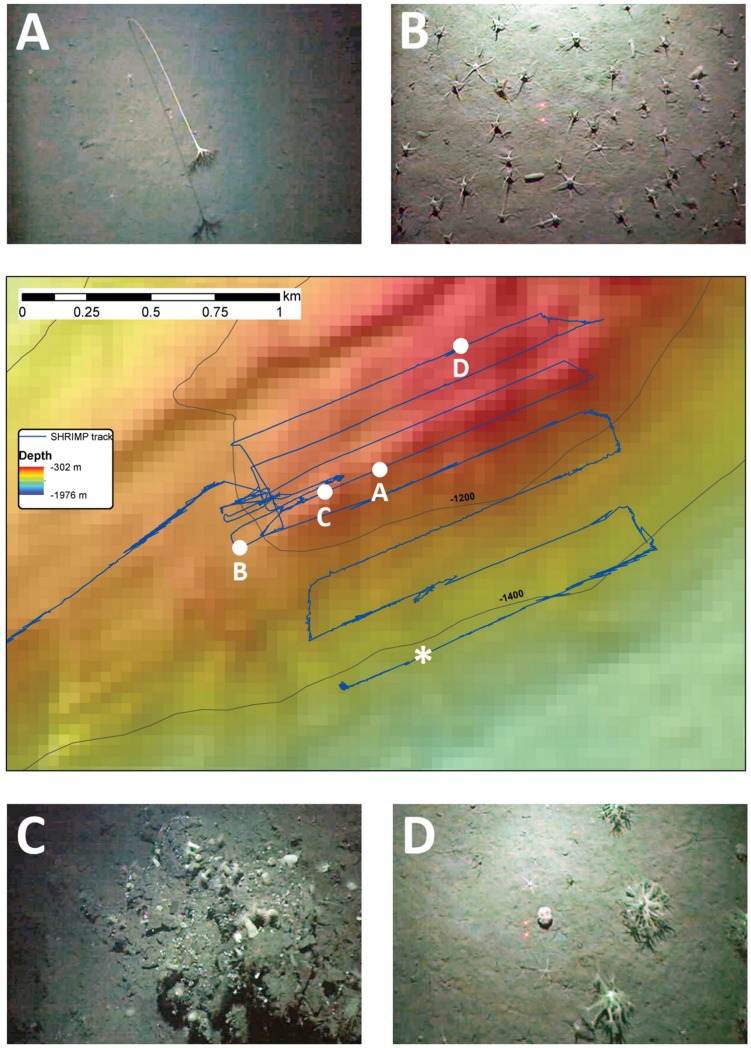
SHRIMP images of benthic fauna at Hook Ridge. (A) *Umbellula* sp. pennatulid and ophiuroids on sedimented seafloor. (B) Aggregation of ophiuroids (∼15 m^−2^) and holothurians on sedimented seafloor. (C) Sea anemones, scleractinians, and sponges on rock outcrop. (D) Soft corals. Red laser dots visible in images are 0.1 m apart. Locations of images are indicated along SHRIMP survey lines on bathymetric map; asterisk denotes location of a reference site away from the hydrothermal area where ophiuroid densities were compared.

Several faunal assemblage types were observed on Hook Ridge from the SHRIMP tow-camera system. Sedimented areas were occupied by taxa including holothurians, asteroids, echinoids, enteropneusts, and pennatulids (Figure 7A). Areas dominated by ophiuroids were also encountered on sedimented seafloor (Figure 7B). Rocky outcrops were occupied by anemones, octocorals, and solitary scleractinians (Figure 7C). Within the graben-like depression on the crest of the ridge, areas dominated by soft corals were also present (Figure 7D).

There was also no visible increase in the abundance of fauna in proximity to the hydrothermally active area. The abundance of ophiuroids nearest to the hydrothermally active area (∼15 m^−2^; Figure 7B) was no greater than that of another ophiuroid bed away from the area and more than 700 m deeper on the slope of the ridge (∼20 m^−2^; reference site location denoted by the asterisk in Figure 7). Specimens of a siboglinid polychaete, *Sclerolinum* sp., were recovered in megacore samples (MC7) from the hydrothermal area. The morphology and molecular phylogenetics of these specimens are being analysed in a separate study by Adrian Glover at the Natural History Museum in London, but the genus is also known from non-vent environments [19], [20]. *Sclerolinum* is considered to represent a monogeneric clade (Monilifera) of siboglinids that do not exhibit the further habitat specialisation of vestimentifera [34]. No recognisable specialist vent fauna were observed in the vicinity of the seafloor hydrothermal sources at Hook Ridge in the SHRIMP imagery.

At Middle Sister, the crest and slope of the ridge were characterised by exposed basalt including eroded and sedimented pillows, giving way to sedimented seafloor to the north. In spite of extensive seafloor surveys, no evidence of hydrothermal activity in the form of shimmering water or bacterial mats was observed. Fauna occupying rock substrata were dominated by sponges and anemones on the slope of the ridge, with octocorals and solitary scleractinians occurring in greater abundance at the ridge crest. Areas of sedimented seafloor were dominated by ophiuroids and asteroids. No recognisable specialist vent fauna were observed during the SHRIMP survey of Middle Sister.

### Sediment and Pore Fluid Characteristics

**Figure 8:**
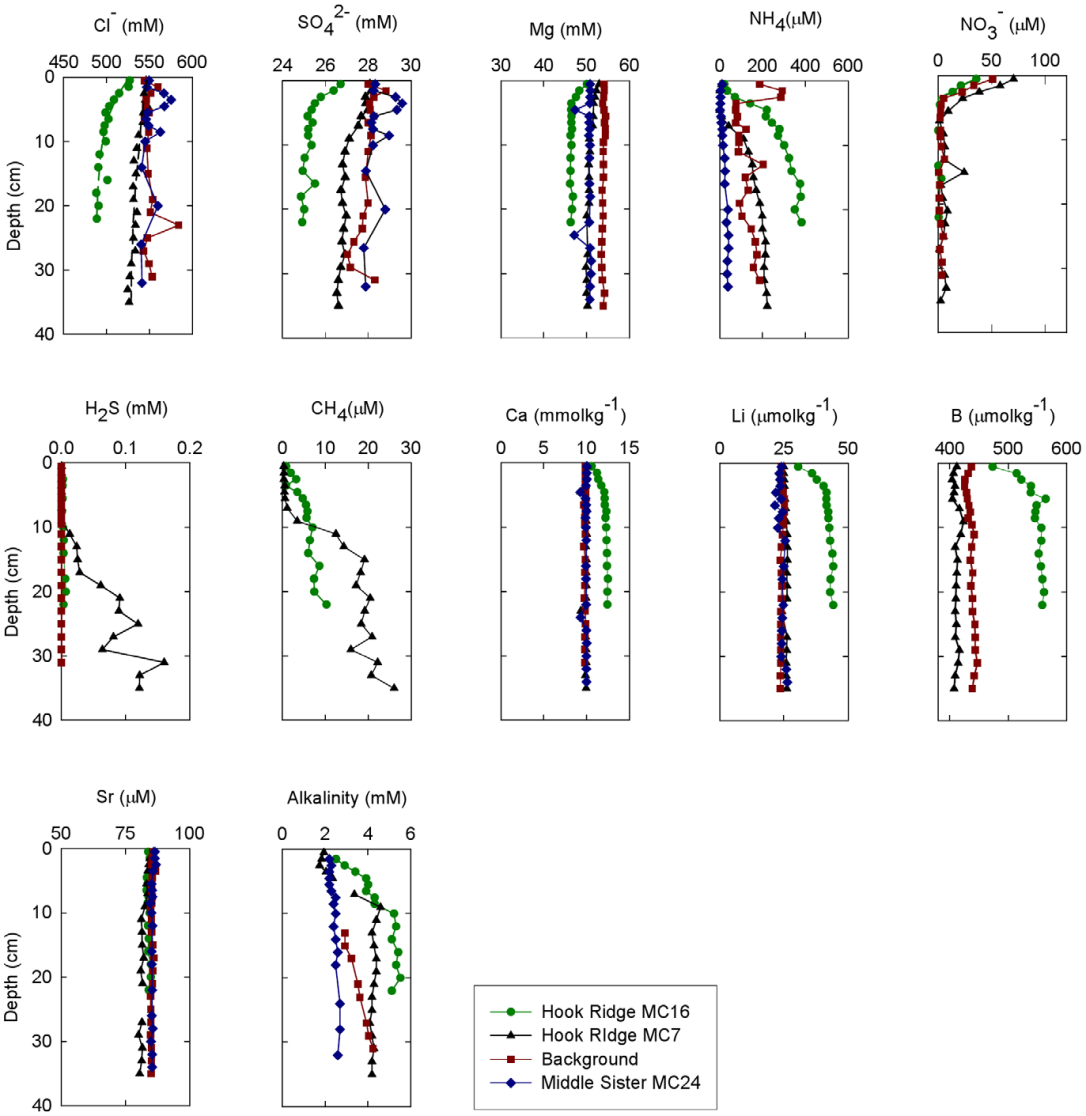
Interstitial fluid profiles for cores from the background site, Hook Ridge and Middle Sister. Measured concentration-depth pore fluid profiles of Cl^−^, SO_4_^2−^, Mg, NH_4_^+^, NO_3_^−^, H_2_S, CH_4_, Li, Ca and B in hydrothermally-influenced Hook Ridge cores MC7 and MC16 and non-hydrothermally-influenced background core and Middle Sister core MC24. Dashed lines represent Cl^−^ profiles in MC7 and MC16 calculated using a one-dimensional diffusion-advection model; best fits were obtained by applying flow velocities of 9 cm yr^−1^ and 33 cm yr^−1^, respectively.

Sediment surface temperature at Hook Ridge, Middle Sister and the background site, measured onboard the RRS *James Cook* immediately following core retrieval, was similar to ambient (∼ 0°C); this is in contrast to previous reports of temperatures (measured on board) of up to 48.5°C from the same area of Hook Ridge [21]. Concentration-depth profiles of dissolved Cl^−^, SO_4_^2−^, Mg, NH_4_^+^, NO_3_^−^, H_2_S, CH_4_, Li, Ca, B and alkalinity are presented in Figure 8. The pore fluid Sr isotope composition (^87^Sr/^86^Sr) in the deepest sample from core MC16 (22 cm) was 0.708776±0.000012.

Overall, concentration-depth profiles of diagenetic indicators are similar at all sites. For example, the down-core increase in alkalinity results from microbial respiration, and the rapid decrease in NO_3_^−^ and the concomitant increase in NH_4_^+^ indicate bacterial nitrate reduction. However, down-core profiles of Cl^−^, SO_4_^2−^ and H_2_S in Hook Ridge cores are distinct from the non-hydrothermally influenced cores at the reference site and at Middle Sister. At Hook Ridge, downcore depletions in Cl^−^ content (4 and 7% in cores MC7 and MC16, respectively) are consistent with upward advection of a low-chlorinity hydrothermal end-member; advection rates estimated from one-dimensional advection-diffusion modelling of the Cl^−^ profiles in MC7 and MC16 are 9 cm yr^−1^ and 33 cm yr^−1^, respectively, comparable to previous estimates at Hook Ridge of 18 to 24 cm yr^−1^ [18]. In the background core and at Middle Sister, SO_4_^2−^ content does not change significantly with depth, indicating that sulfate reduction is negligible in the top ∼40 cm of the sediment column. In contrast, at Hook Ridge, SO_4_^2−^ in the deepest samples is depleted by 5% (MC7) and 11% (MC16) relative to average seawater concentrations (28 mM). By comparison to the reference core, it can be assumed that bacterial sulfate reduction is insignificant in the Hook Ridge cores and, therefore, sulfate depletion in MC7 and MC16 and elevated H_2_S in MC7 can be attributed to hydrothermal influence. Methane concentrations increase downcore, up to 26 µmol l^−1^ and 10 µmol l^−1^ in cores MC7 and MC16, respectively (Figure 8). Because microbial methanogenesis occurs only when the sediment becomes highly reducing [35], microbial methane production can be ruled out, suggesting a non-biotic hydrothermal origin for methane in the Hook Ridge cores.

Downcore pore fluid Mg does not vary with depth in the background core (MC3) and at Middle Sister (MC24), but is depleted by 5% and 8% at the base of cores MC7 and MC16, respectively (Figure 8). In the solid phase, the downcore Mg/Ti ratio is relatively constant at the background site (decreasing from 7.7 to 7.4 on a molar basis), while at Hook Ridge the molar ratio decreases from 7.3 to 6.6 and from 8.5 to 5.8 in cores MC7 and MC16, respectively. This suggests Mg desorption from the sediment matrix in the hydrothermally-influenced cores, similar to previous observations at Hook Ridge [24], and results in non-conservative mixing between seawater and the hydrothermal end-member.

## Discussion

### Location and Visualization of Fluid Seepage

The discharge of hot, chemically-altered fluids produces plumes which rise above the seafloor, eventually dispersing over large distances by ocean currents, and the detection of physical and chemical anomalies in the water column associated with buoyant and neutrally-buoyant plumes provides a useful tool to locate hydrothermal vents on the seafloor. At Hook Ridge, the low-lying plume of cool, transparent and chemically reducing fluid identified by CTD profiling (Figures 5A–C) is not consistent with focused hydrothermal discharge from a point source, but rather indicates diffuse flow at low temperatures with no accompanying precipitation. Similar low-lying hydrothermal plumes of reducing fluid, occasionally also exhibiting small temperature and turbidity anomalies, have been observed in other areas of diffuse flow, for example at the Gakkel Ridge in the Arctic Ocean [36] and at the Galàpagos Spreading Centre [37].

Low chlorinity pore fluids demonstrate the presence of a low salinity end-member flowing through the substrate, and the plume of shimmering water bears testimony to the seepage of fluids with different salinity and/or temperature characteristics compared to seawater. Although active high temperature venting was not observed, the presence of a small (∼ 2 m) relict mineralized chimney at Hook Ridge (Figure 6) and previous reports of warm, buoyant and turbid water column plumes [4], [21] suggest that high temperature hydrothermal discharge may have occurred previously. At Middle Sister, small E_h_ anomalies constitute some evidence of hydrothermal release; however, the apparent absence of seafloor manifestations of fluid release makes this difficult to verify. The absence of water column plumes at The Axe suggests that hydrothermal discharge was not occurring from this edifice.

### Influence of Hydrothermal Circulation on Pore Fluid Geochemistry

The concentration-depth profiles presented here (Figure 8) suggest that at Hook Ridge, pore fluid composition is affected by both hydrothermal fluid flow and organic matter diagenesis and, in this respect, are similar to other sediment-hosted hydrothermal systems such as the Guaymas Basin, Gulf of California [38] and the Wakamiko Crater, Kagoshima Bay [39], [40]. In contrast, concentration-depth profiles at Middle Sister and the background site provide a record of organic matter diagenesis but no indication of hydrothermal influence.

End-member hydrothermal solutions are usually assumed to contain no dissolved Mg, since water-rock reactions in the recharge zone quantitatively remove Mg from solution to form Mg-OH silicates [41], and no dissolved sulfate, which is depleted due to precipitation of anhydrite at temperatures above 150°C at high pressures [42]. Therefore, the downcore Mg and SO_4_^2−^ distributions (Figure 8) are consistent with the upwelling of a hydrothermal end-member and mixing with seawater. The distinct pore fluid ^87^Sr/^86^Sr isotope ratio (0.708776±0.000012) relative to modern seawater (^87^Sr/^86^Sr ≈ 0.70918; [43]) can be explained by mixing between hydrothermal fluid enriched in non-radiogenic ^86^Sr, sourced from volcanic rocks (^87^Sr/^86^Sr in Bransfield Strait volcanic rocks varies between 0.7026–0.7037; [44]), and seawater. The low Cl^−^ content of the upwelling fluid is inferred to arise from phase separation of the hydrothermal fluid in the shallow subsurface [21], [24]. Furthermore, hydrothermal solutions are typically enriched in Ca, Li and B [1] and, consequently, elevated Ca, Li and B contents in MC16 likely result from upwelling of the hydrothermal end-member.

Theoretically, the hydrothermal component in the pore fluid samples can be estimated from simple mixing between hydrothermal fluid and seawater, assuming a hydrothermal end-member devoid of Mg; such models are conventionally used to determine the composition of high temperature black smoker fluids by correcting for the mixing that occurs during sampling [45]. However, the assumption of a zero-Mg hydrothermal end-member is not always valid, since Mg can be leached back into solution in the presence of highly acidic fluids [46], and because phase separation may result in Mg fractionation between the liquid and vapour phases [46]. Furthermore, the downcore decrease in Mg/Ti ratios in the solid phase indicates desorption of Mg^2+^ ions from the substrate, possibly due to elevated pore water alkalinity [47], cation exchange with NH_4_^+^ ions formed by organic matter degradation [48], or desorption as a result of hydrothermal fluid flow through the sediment [38]. Because of non-conservative mixing, the assumption of zero Mg in the hydrothermal end-member is not applicable, and Mg cannot be used to estimate its composition in this case. In contrast, sulfate appears to behave conservatively, and a simple mixing model (with zero and 28.9 mmol kg^−1^ SO_4_^2−^ in the hydrothermal and seawater end-members, respectively) yields a hydrothermal component of 8% and 14% in the deepest samples of cores MC7 and MC16, respectively (compared with 24% measured previously in the same region; [24]).

**Figure 9:**
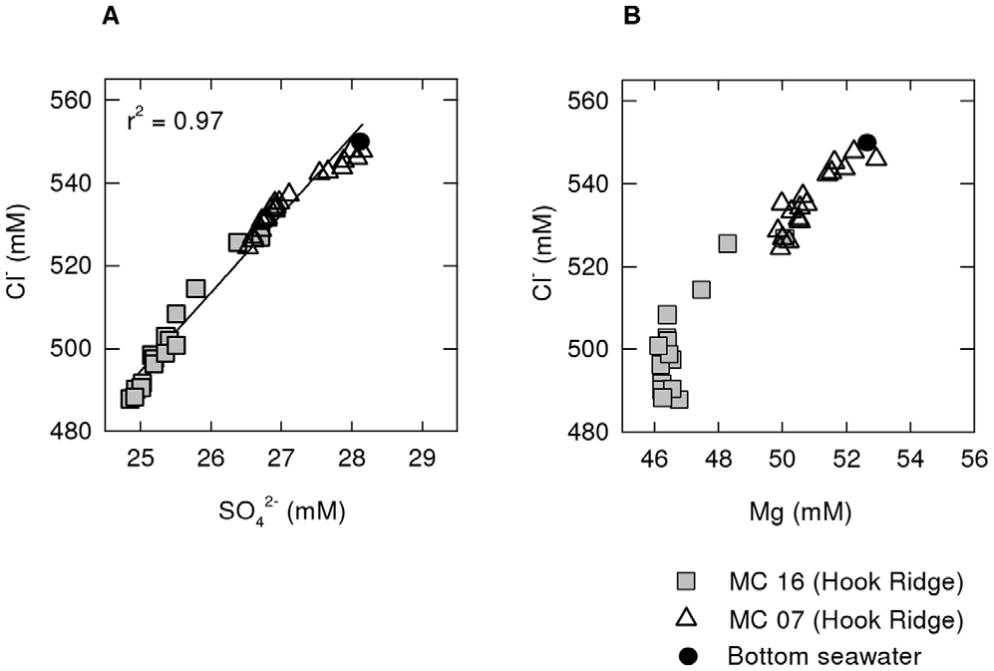
Correlation plots of pore fluid constituents in Hook Ridge cores. (A) Plot of [Cl^−^] and [SO_4_^2−^] data for cores MC7 and MC16. The [Cl^−^] in the hydrothermal end-member, determined by extrapolation the linear regression to zero [SO_4_^2−^], is 22 mM. (B) Plot showing the relationship between pore water [Cl^−^] and [Mg] in cores MC7 and MC16.

Downcore depletion in dissolved Cl^−^ content in cores MC7 and MC16 is consistent with the upwelling of a Cl-depleted fluid, and is also in agreement with previously reported evidence for phase separation and subsequent transport and mixing of the vapour phase at Hook Ridge [21], [24]. The end-member Cl^−^ can be estimated using a simple mixing model of hydrothermal and seawater end-members, using the mixing ratio determined from the SO_4_^2−^ concentration-depth profile. Using the deepest sample from MC16 (which consists of 14% of a hydrothermal component at 22 cm depth) and a bottom water Cl^−^ of 540 mM (seawater salinity 34.5 at 1188 m water depth), the calculated end-member Cl^−^ is 171 mM. Alternatively, extrapolation of the linear regression between [Cl^−^] and [SO_4_^2−^] using data from cores MC7 and MC16 (Figure 9A) results in an end-member [Cl^−^] of 22 mM. These values are comparable to previous estimates of 1–84 mM [24]; the wide range is due to the sensitivity of this method to small changes in end-member concentrations. Because Mg does not behave conservatively in this setting (see discussion above), the relationship between Cl^−^ and Mg is not linear (Figure 9B) and, therefore, a plot of Mg against Cl^−^ cannot be used to estimate the extent of mixing between hydrothermal and seawater end-members and, consequently, the end-member composition.

### Conclusions

Using a combination of water column, seafloor, and sediment core analyses, it has been shown that low-temperature hydrothermal discharge of phase separated fluids significantly diluted by seawater occurs through Hook Ridge sediments and is observed as shimmering water issuing from the sediment and from mineralised structures on the seafloor. Despite extensive searches, there was no evidence of discharge at high temperatures from the investigated areas in the Central Bransfield Strait. No recognisable vent-endemic fauna were observed in the surveyed areas; this has been previously attributed to low sulfide concentration and fluxes [18], but may also be related to the ephemeral nature of the hydrothermal activity in this setting. There was no conclusive evidence of hydrothermal circulation at Middle Sister and The Axe.
